# PTEN regulates spindle assembly checkpoint timing through MAD1 in interphase

**DOI:** 10.18632/oncotarget.20532

**Published:** 2017-08-24

**Authors:** Yu Liu, Xiao Du, Shuting Zhang, Yang Liu, Qiaoling Zhang, Qi Yin, Michael A. McNutt, Yuxin Yin

**Affiliations:** ^1^ Institute of Systems Biomedicine, Department of Pathology, School of Basic Medical Sciences, Beijing Key Laboratory of Tumor Systems Biology, Peking-Tsinghua Center for Life Sciences, Peking University Health Science Center, Beijing 100191, China

**Keywords:** PTEN, MAD1, spindle assembly checkpoint, mitosis, genome stability

## Abstract

The spindle assembly checkpoint (SAC) restrains anaphase progression to ensure all chromosomes attach properly to the spindle. Although SAC timing has been extensively investigated in mitosis, its mechanism of regulation in interphase is unclear. We report that PTEN functions as a crucial activator of SAC timing and protects chromosome segregation under both spindle poison treated and untreated conditions. We show that PTEN physically interacts with MAD1 and promotes its dimerization and localization in the nuclear pore. Consequently, PTEN is important for the formation of the mitotic checkpoint complex (MCC) in interphase. We propose PTEN/MAD1 signaling is essential for maintenance of SAC timing and chromosome integrity.

## INTRODUCTION

The spindle assembly checkpoint (SAC) prevents chromosome separation until each chromosome is properly attached to the spindle. SAC timing is critical for chromosome stability, as its anaphase delay signal allows a critical interval of time that ensures faithful kinetochore-microtubule (KT-MT) attachment [1, 2]. Current studies have concluded that the SAC is directly coupled to KT-MT attachments by its promotion of the mitotic checkpoint complex (MCC) during mitosis [3–5]. However, it must be emphasized that MAD1 also contributes to SAC timing through shuttling nuclear transport [6, 7], and assembly of a pre-mitotic anaphase inhibitor in interphase [8]. MAD1 cycles between the nuclear pore complex (NPC) and the kinetochore MCC during the cell cycle, and its interphase localization at the NPC is required for generation of a sufficient SAC timing interval [9]. However, the underlying mechanism of interphase MAD1 modulation is unknown.

PTEN is recognized as an important tumor suppressor. In addition to its well-characterized regulation of cell survival, PTEN is also involved in global maintenance of genome stability [10–13]. To gain insight into additional mechanisms which underlie PTEN protection of the genome, we investigated the PTEN function in protection of SAC timing. Chromosome instability in *Pten* null MEFs is phenotypically similar to that seen in *Mad1* depletion [14]. This observation caught our attention, and raised the possibility that PTEN participates in MAD1 related preservation of chromosome integrity.

## RESULTS

### PTEN maintains SAC timing

**Figure 1:**
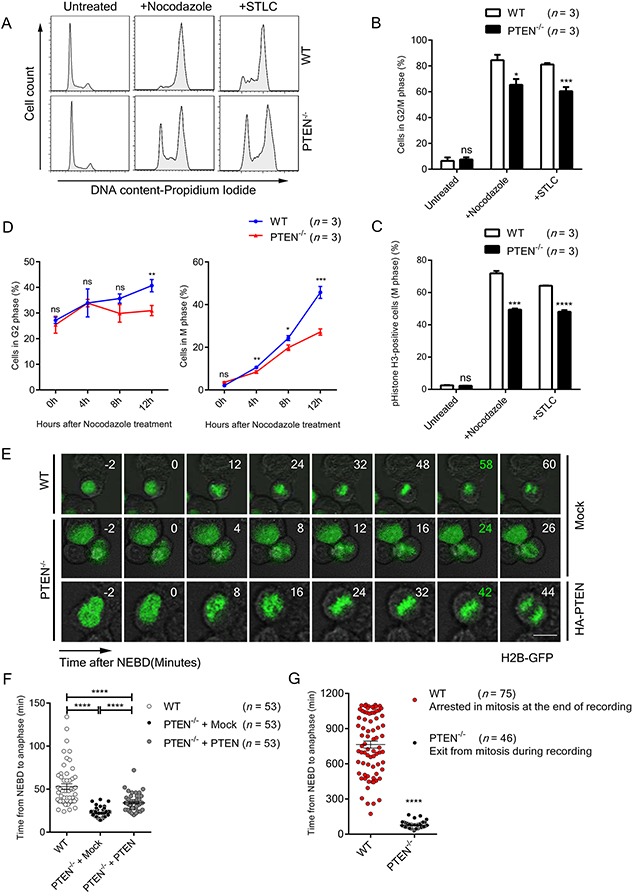
PTEN maintains SAC timing **(A)** FACS profiles of Propidium Iodide (PI) stained WT and PTEN^−/−^ HCT116 cells. Cells were treated with indicated spindle poison for 12 hours. **(B)** Flow cytometry analysis of WT and PTEN^−/−^ HCT116 cell cycle. Cells were treated with indicated spindle poison for 12 hours before FACS assay. Percentage of G2/M phase cells was analyzed from FACS profiles fitted with the “Dean-Jett-Fox” model. *n*, number of independent FACS assays (~10 000 cells were analyzed each time). **(C)** FACS profiles of pHistone-H3 labeled (M phase) WT and PTEN^−/−^ HCT116 cells. Cells were treated with indicated spindle poison for 12 hours and labeled with pHistone-H3. *n*, number of independent FACS assays (~10 000 cells were analyzed each time). **(D)** FACS profiles of G2 and M phase for WT and PTEN^−/−^ HCT116 cells. Cells were treated with nocodazole, collected at indicated time points and labeled with pHistone-H3 and PI. Percentage of cells in G2 phase and M phase were analyzed as described in B, C. *n*, the number of independent FACS assays (~10 000 cells were analyzed each time). (E) Live cell imaging of WT and PTEN^−/−^ HCT116 cells. Cells expressing H2B-GFP were tracked during unperturbed mitosis. Time ‘0’ denotes NEBD. Time marked in green denotes anaphase onset. Scale bars, 10 μm. **(F)** – **(G)** Statistical analysis of SAC timing. The interval from NEBD to anaphase was determined with time-lapse recordings after GFP-H2B transfection of indicated cells. Cells were treated with nocodazole before recording in **G**. *n*, number of mitotic cells counted. Values in **B, C, D** represent mean ± SD, and values in **F** represent mean ± SEM. Statistical significance was determined by unpaired t-test, ns denotes non-significant, ^*^P<0.05, ^**^P<0.01, ^***^P<0.001, ^****^P<0.0001. See also Supplementary Figure 1.

PTEN is involved in the maintenance of centromere stability and chromosome integrity [11, 13, 15]. This raised the possibility that PTEN also participates in mitotic cell cycle regulation and influences chromosome segregation. The role of PTEN in the cell cycle was first evaluated with flow cytometry. Unperturbed PTEN^−/−^ HCT116 cells showed a cell cycle distribution similar to WT cells (Figure 1A, top panel, Figures 1B and 1C). However, under treatment with nocodazole or the EG5 inhibitor S-trityl-L-cysteine (STLC), HCT116 cells were effectively arrested at M phase, while the M phase synchronization ratio of PTEN null cells was significantly reduced compared with WT cells (Figure 1A lower panel, Figures 1B and 1C).

To determine whether this phenomenon is caused by SAC arrest dysfunction or by defects in other stages of the cell cycle, we tracked the proportional change of G2 and M phase cells after nocodazole treatment in WT and PTEN^−/−^ HCT116 cells. As shown in Figure 1D and Supplementary Figure 1A, there was a significant reduction in M phase in PTEN^−/−^ HCT116 cells compared to WT cells, while the G2 phase cells exhibited no meaningful difference during the blocking process, indicating that loss of PTEN does not bring about delayed G2/M entry. In addition, we tracked the cyclin levels and cell cycle patterns of WT and PTEN^−/−^ HCT116 cells after thymidine block release. Consistent with the results above, the protein level of Cyclin B1 in PTEN null cells showed no significant difference as compared to WT cells, suggesting G2/M transition efficiency is similar in these two cell lines (Supplementary Figure 1B). Moreover, longer cell cycle duration was likely not the reason for impaired nocodazole arrest in PTEN null cells. As shown in Supplementary Figure 1C, both WT and PTEN^−/−^ HCT116 cells recovered to normal cell cycle patterns 12 hours after thymidine block release. We thus conclude that it is SAC function rather than other stages of the cell cycle that are dampened by loss of PTEN. As for the AKT pathway, treatment with the PI3K inhibitor LY_294002_ did not restore the M phase synchronization ratio in PTEN^−/−^ HCT116 cells (Supplementary Figure 1D), arguing PTEN regulation of SAC arrest is independent of the AKT pathway.

To investigate the relationship of PTEN and SAC timing, the interval from nuclear envelope break down (NEBD) to anaphase was evaluated with time-lapse recording. Under normal conditions, anaphase occurred on an average of 53 min after NEBD in HCT116 cells, in sharp contrast to 22 min in PTEN^−/−^ HCT116 cells, and this change was partially reversed by re-expression of PTEN (Figure 1E and Figure 1F, left lane vs. middle and right lane). We expanded upon this observation with nocodazole treatment, and found that the PTEN null cells cannot be arrested in mitosis (Figure 1G). These results demonstrate that both spontaneous and conditional SAC override occur as consequences of PTEN ablation.

### PTEN preserves chromosome integrity

**Figure 2:**
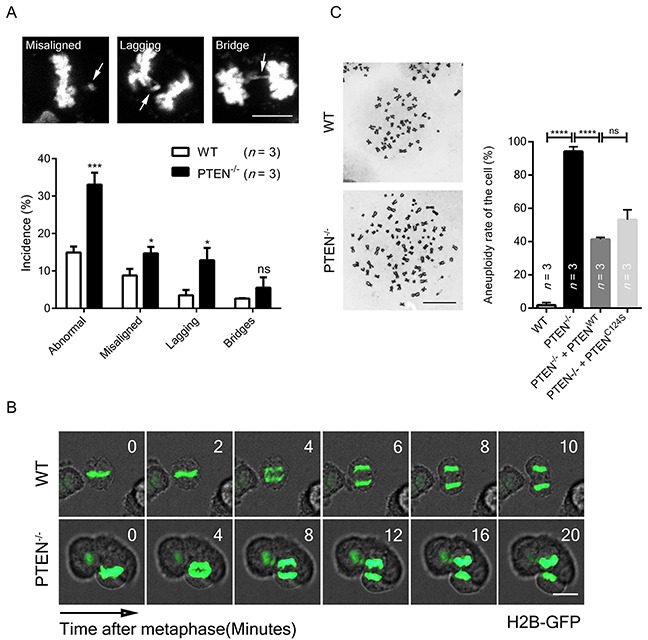
PTEN preserves chromosome integrity **(A)** Chromosome segregation analysis of WT and PTEN^−/−^ HCT116 cells. Standards for misaligned, lagging and bridging chromosome are shown. White arrows indicate mis-segregation. *n*, number of independent experiments (36 ~ 40 cells per experimental group). Scale bar, 10 μm. **(B)** Live cell imaging of WT and PTEN^−/−^ HCT116 cells. Cells expressing H2B-GFP were tracked during unperturbed mitosis. Time ‘0’ denotes anaphase onset. Scale bar, 10 μm. **(C)** Aneuploidy analysis of WT and PTEN^−/−^ HCT116 cells. Cells were transfected with indicated FLAG-tag overexpression plasmid for 48 hours before observation. Representative images of chromosome spreads are shown. *n*, number of independent experiments (34 ~ 36 cells per experimental group). Scale bar, 10 μm. Values for all data are presented as mean ± SD. Statistical significance was determined with the unpaired t-test, ^*^P<0.05, ^**^P< 0.01, ^****^P<0.0001.

To determine whether mitosis was impaired in the absence of PTEN, abnormal chromosome incidences were visualized by immunofluorescence, and severe chromosome mis-segregation was found in metaphase and anaphase (Figure 2A, top panel). Significant increases in chromosome misalignment, lagging and bridging were also identified by statistical analysis (Figure 2A, lower panel), which is consistent with the finding that PTEN^−/−^ cells displayed a longer anaphase than normal cells (Figure 2B).

To determine whether chromosome integrity is compromised by such inaccurate chromosome segregation, karyotyping was employed, and significantly increased aneuploidy was observed in PTEN^−/−^ cells (Figure 2C, lane 1 vs. lane 2). This alteration was partially rescued by re-expression of either WT PTEN or C124S PTEN (Figure 2C, lane 3 and lane 4), arguing that PTEN function in chromosome segregation is phosphatase activity independent. These results indicate PTEN is necessary for proper chromosome segregation, and its influence may be exerted through protection of SAC.

### PTEN interacts with MAD1 in interphase

**Figure 3:**
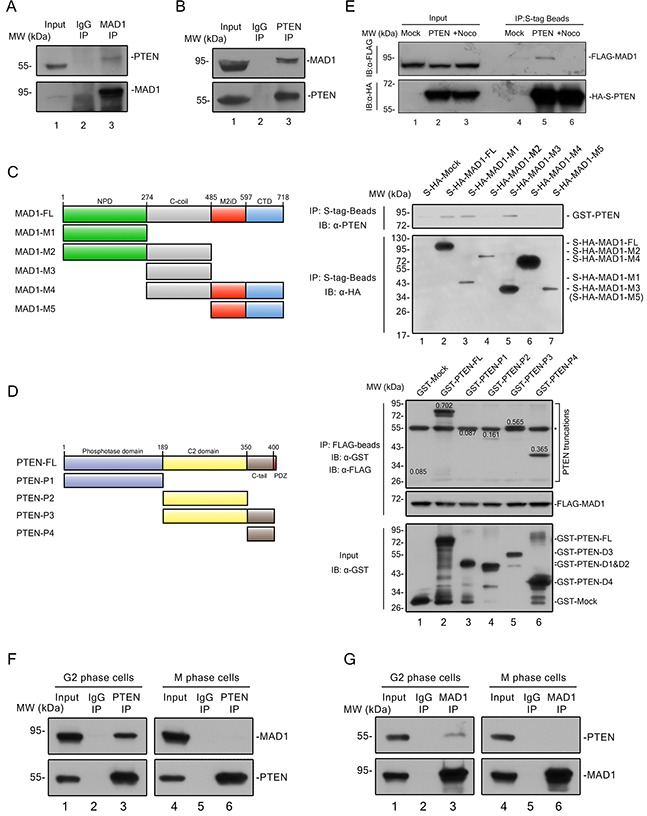
PTEN interacts with MAD1 in interphase **(A)** – **(B)** Immunoprecipitation of PTEN and MAD1. PTEN or MAD1 was immunoprecipitated from HCT116 cell extracts and immunoblotted for MAD1 or PTEN. IgG, immunoglobulin G. **(C)** *In vitro* binding assay. Left panel, overview of MAD1 and its truncations. Right panel, S-HA tagged MAD1 and its truncations were pulled down with S-tag beads, and tested for binding to purified GST-PTEN. **(D)** *In vitro* pull-down assay. Left panel, overview of PTEN and PTEN truncations. FLAG tagged MAD1 was pulled down with FLAG-beads, and tested for binding to purified GST-Mock, GST tagged PTEN and related truncations. Number on each PTEN truncation band denotes its relative intensity ratio compared to the input band, which indicates its binding ability with MAD1. The asterisk designates a non-specific band of GST antibody. **(E)** S tag pull down analysis. Exogenous S-HA-PTEN and FLAG-MAD1 were transfected into HCT116 cells. PTEN was pulled down by S-tag beads and detected with a FLAG antibody for MAD1 with or without nocodazole treatment. Mock, S-HA tagged mock protein. **(F)** – **(G)** Conditional immunoprecipitation of PTEN and MAD1. G2 phase cells were acquired after 12 hours of thymidine block and 8 hours of release, M phase cells were acquired by mitotic shake off after 12 hours of nocodazole treatment. PTEN or MAD1 was immune precipitated from G2 or M HCT116 cells and immunoblotted for MAD1 or PTEN. IgG, immunoglobulin G. See also Supplementary Figures 2 and 3.

MAD1 has previously been shown to play a critical role in SAC timing and regulation of chromosome stability [8, 9], and we confirmed this with a TALEN knock-out cell line (Supplementary Figures 2A and 2B). MAD1 was on our PTEN pull down list (Supplementary Figure 3A, lane 2 and Supplementary Figure 3B, dark-green and orange dots), and this raised the possibility that PTEN controls SAC at least in part through MAD1. Endogenous interaction of these molecules was verified by IP-Western experiments (Figure 3A, lane 3 vs. lane 2 and Figure 3B, lane 3 vs. lane 2). Truncation pull down analysis confirmed that PTEN binds to the MAD1 nuclear pore-targeting domain (NPD) and C-coil domain (Figure 3C, lane 1~3), and that the C2 and C-tail domains of PTEN are required for MAD1 interaction (Figure 3D, lane 5 and 6). We also generated several PTEN mutants to mimic PTEN phosphorylation on the C2 and C-tail domains, but no significant difference was observed in the interaction of these mutants with MAD1 (Supplementary Figure 3C). This argues interaction of PTEN and MAD1 may not depend on the phosphorylation status of PTEN.

As MAD1 has been reported to function in both interphase and mitosis, we sought to determine the phase in which association of PTEN and MAD1 occurs. Physical interaction of exogenous PTEN and MAD1 was abolished by nocodazole treatment (Figure 3E, lane 5 vs. lane 6), and this finding was confirmed by separate endogenous immunoprecipitations in G2 and M phase HCT116 cells (Figures 3F and 3G). This suggested interaction of PTEN and MAD1 occurs in interphase.

### PTEN promotes MAD1 dimerization and MCC assembly in interphase

**Figure 4:**
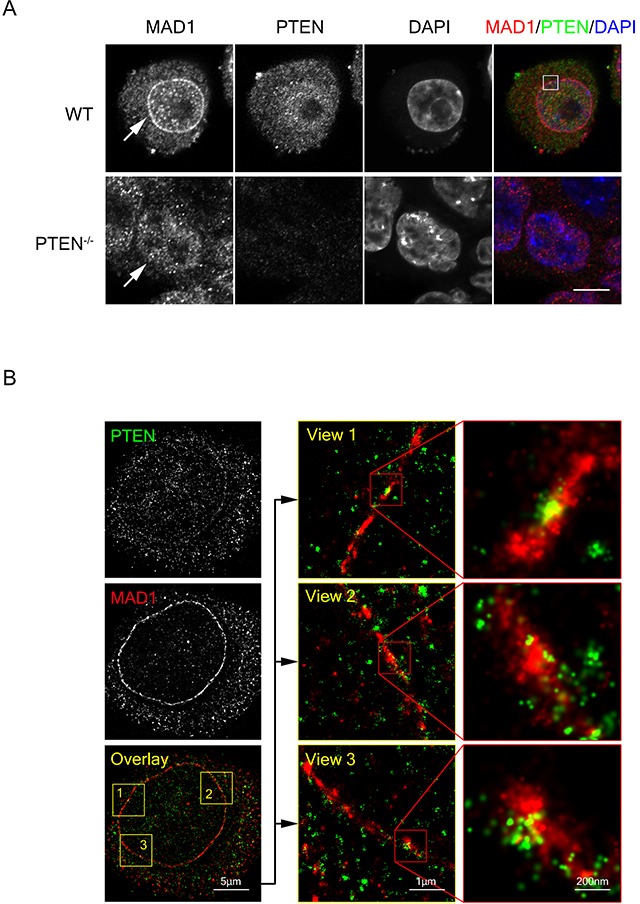
PTEN co-localizes with MAD1 at the nuclear envelope **(A)** Immuno-fluorescence staining of fixed WT and PTEN^−/−^HCT116 cells. Cells were stained for MAD1 (red), PTEN (green) and DNA (blue). Single z sections are shown with scale bars in 10 μm. White square denotes co-localization point of MAD1 and PTEN. White arrows denote nuclear envelope localization of MAD1. **(B)** STORM super resolution image of PTEN and MAD1. Fixed HCT116 cells were stained for PTEN (green) and MAD1 (red). Single z sections of co-localization views are shown. PTEN exhibited a circle pattern similar to MAD1. See also Supplementary Figures 4 and 5.

**Figure 5:**
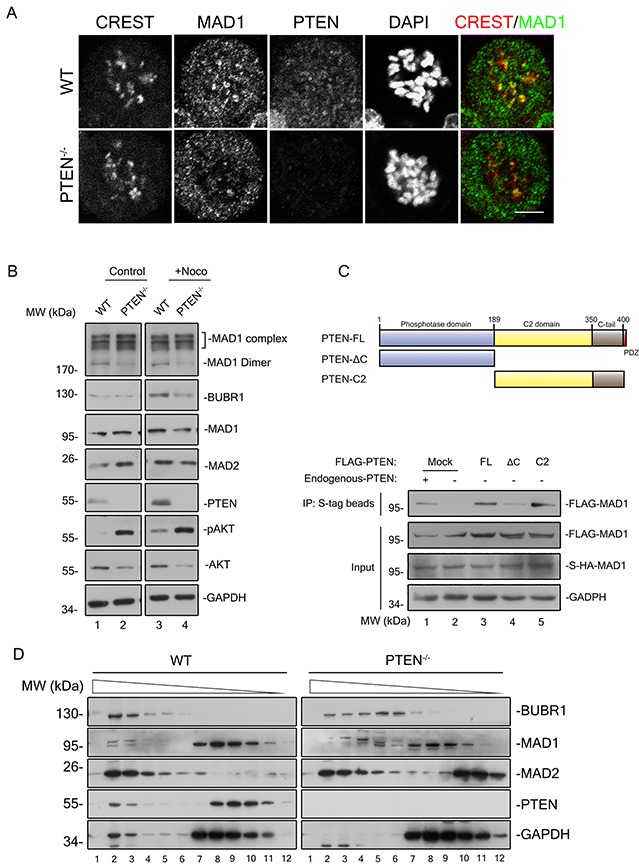
PTEN promotes MAD1 dimerization and MCC assembly in interphase **(A)** Immunofluorescence staining of fixed WT and PTEN^−/−^ HCT116 cells. Cells were treated with nocodazole and MG132 for 90 minutes prior to staining for CREST (red), MAD1 (green), PTEN (magenta) and DNA (blue). Single z sections are shown with scale bars of 5 μm. **(B)** Immunoblotting of MAD1 and indicated molecules in interphase and mitosis from WT and PTEN^−/−^ HCT116 cell extracts (sample prepared without heat treatment). Black square indicates MAD1 protein complexes. **(C)** S-Tag pull down analysis of MAD1 dimer. Overview of PTEN and its truncations are shown. S-HA-MAD1 and FLAG-MAD1 were co-transfected into PTEN^−/−^HCT116 cells with or without FLAG-PTEN. S-HA-MAD1 was pulled down by S-tag beads prior to evaluation of protein binding with indicated antibodies. The intensity of the FLAG-MAD1 IP band indicates its ability to form dimers with S-HA-MAD1. Mock, FLAG tagged mock protein. **(D)** Gel filtration chromatography of WT and PTEN^−/−^ HCT116 cells. Indicated MCC components were blotted in interphase extracts of indicated cell lines after gel filtration chromatography. Chromatography separates components of different molecular weights.

To further evaluate the role of PTEN in MAD1 regulation, we examined the subcellular location of PTEN and MAD1 in both interphase and mitosis. Partial co-localization of PTEN and MAD1 was observed at the nuclear envelope in interphase, both with immunofluorescence and with STORM super resolution imaging analysis (Figure 4A, upper panel, Figure 4B and Supplementary Figure 4). As MAD1 localizes primarily at the mitotic kinetochores under treatment with spindle poisons, we treated cells with nocodazole and found that MAD1 kinetochore localization in mitosis is independent of the presence or absence of PTEN (Figure 5A), suggesting that PTEN is involved in promotion of MAD1 during interphase instead of in mitotic phase. This is consistent with the results of our previous binding analysis that indicated PTEN interacts with MAD1 only in interphase (Figure 3E). These results suggest PTEN plays an important role in promoting MAD1 in interphase.

To investigate the mechanism by which PTEN regulates SAC through MAD1, we sought to evaluate molecular level changes. Previous studies have determined that the interaction between monovalent MAD1 and MAD2 is much weaker than the interaction of dimerized MAD1 and MAD2 [16]. The capacity of MAD1 for dimerization is thus crucial for SAC function. PTEN depletion resulted in a significant decrease of MAD1 dimerization in both interphase and mitotic phase (Figure 5B, lane 1 vs. lane 2, lane 3 vs. lane 4 and Figure 5C, lane1 vs. lane 2), and this decrease was reversed by re-expression of full-length PTEN or the PTEN C2 region (Figure 5C, lane 3 & 5 vs. lane 2). This offers a mechanistic explanation for PTEN participation in SAC regulation. Consistent with the result of dampened MAD1 dimerization, the ability of MAD1 for NPC localization was also reduced in PTEN^−/−^ cells (Figure 4A and Supplementary Figure 5).

As interphase localization of MAD1 on the NPC is critical for pre-assembly of the MCC [9] which in turn is responsible for anaphase inhibition, MCC assembly ought to be affected in PTEN^−/−^ cells. The major components of the MCC were evaluated with gel filtration chromatography analysis, and it was a surprise to find a large proportion of both MAD2 and BUBR1 reside in PTEN null cells as monomers (Figure 5C, right panel, lane 5-8 and lane 10-12), in sharp contrast to HCT116 cells where most of these components reside in protein complexes (Figure 5D, left panel, lane 2-4), The phosphorylation status of PTEN was also monitored during this analysis, and we found that phosphorylated PTEN is mainly monomeric (Supplementary Figure 6). This indicates that it is the non-phosphorylated, active form of PTEN which co-migrates with MCC. These results argue that PTEN is necessary for promotion of MAD1 and MCC in interphase.

## DISCUSSION

Comprehensive protein network analysis provides an exceptional opportunity to demonstrate the interplay of different pathways and their vital functions on a global scale [17–19]. Although PTEN is one of the most important tumor suppressors, it is recognized that PTEN also orchestrates multiple fundamental cellular processes including cell proliferation and cell motility [20, 21]. Moreover, relationships between PTEN and mitotic participants such as PLK-1 and Aurora-A have been demonstrated [22, 23], which raises the possibility that PTEN may be involved in mitotic regulation [17–19]. The data presented in this study demonstrate MAD1 is associated with PTEN in mitotic cell cycle regulation. For the first time, we demonstrate the essential role of PTEN in protection of SAC timing, which in turn results in protection of chromosome stability.

It is well recognized that PTEN guards against oncogenic processes both through cytoplasmic and nuclear targets [24]. Based on our observations, PTEN is crucial for prevention of chromosome mis-segregation. In this study, we establish that PTEN is a powerful activator of interphase MAD1, and its dysregulation gives rise to aneuploidy and lagging chromosomes. In addition, PTEN may promote MAD1 through its recruitment function. This supports comprehensive understanding of previously unrecognized mechanisms in PTEN pathways.

It has long been recognized that there are substantial amounts of MAD1 at the NPC in interphase [25, 26], but its significance of MAD1 localization in SAC timing regulation has not been appreciated. It is generally believed that localization of MAD1 at the kinetochore is required for this protein to play a role in SAC coupled KT-MT attachment during mitosis [27–29]. Nevertheless, consistent with our findings, a recent study argued that interphase MAD1 is capable of signaling anaphase delay by promoting MCC pre-assembly without altering kinetochore MAD1 function [8, 9]. However, the regulators of interphase MAD1 have heretofore largely been unknown. Our data show that PTEN interacts with MAD1 in interphase, and is important for the balance of its dimerization and MCC formation. In addition, under treatment with spindle poisons, PTEN null cells are less efficiently arrested by SAC, suggesting that SAC timing regulation is potentially of value for assessment of chemotherapy strategy with respect to PTEN status in cancer patients, particularly for cancers carrying PTEN deletions.

In summary, we have demonstrated that PTEN contributes to maintenance of SAC timing. Our results established a mechanism underlying the critical role of PTEN protection of genome stability. Activation of MAD1 in interphase by PTEN with resultant maintenance of SAC timing is critical for chromosomal segregation. We propose that PTEN and MAD1 function as an axis for protection of SAC timing and proper anaphase transition in order to maintain chromosomal integrity and maintain tumor suppressor function.

## MATERIALS AND METHODS

### Cell culture and antibodies

Cells were cultured in MEM supplemented with 10% FBS. The human cancer cell line HCT116 was obtained from the American Type Culture Collection (ATCC) in 2009 and maintained according to ATCC recommendations. This cell line was authenticated by *Beijing Microread Genetics* in November 2016 using STR profiling. The source of the PTEN knock out cell line has been previously described [30].

The following commercial antibodies were used in this study: PTEN (A2B1), PTEN (N19), and MAD1 (H-288) from Santa Cruz; Phosphor-Histone H3 (#3377) from Cell Signaling; CREST autoimmune serum (Immunovision HCT-0100); FLAG (M2-3165) and HA (H3163) from Sigma-Aldrich; BUBR1 (A300-995A), CDC20 (A310-396A) and MAD2L1 (A300-301A) from Bethyl; GADPH, and GST antibodies from Sungene Biotech (Tianjin, China).

Other antibodies which were generated in our laboratory using relevant proteins as antigens included PTEN (mouse monoclonal antibody) and MAD1 (mouse polyclonal antibody).

### Gene targeting

To target *MAD1*, the TALEN knock out system was applied. The cut site within exon1 was chosen to specifically knockout the *MAD1* gene. TALEN vectors targeting the left and right arms were co-transfected into HCT116 cells with PEI (Polysciences, USA). On the third day after transfection, cells were treated with puromycin (2ug/ml) for 3 days and 48 clones were selected and transferred into two 24-well plates without antibiotics. The *MAD1* gene of these clones was analyzed by PCR and DNA sequencing in the 6-well phase. Details of plasmid cloning and expanded protocols can be found in the Extended Experimental Procedures.

### Transfection, immunoprecipitation, and pull-down assays

Transfection was performed using PEI (Polyethyleneimine, Polysciences, Inc.). Immunoprecipitation was performed as previously described (Shen et al., 2007).

Cells transfected with S-HA tagged genes were lysed with cold 0.5% NP40 buffer (pH 8.0-Tris 10 mM, NaCl 150 mM, 0.5% NP40, protease inhibitors) and incubated with S Tag beads 4°C overnight prior to washing with 0.1% NP40 buffer (pH8.0-Tris 10 mM, NaCl 150 mM, 0.1% NP40) followed by SDS-PAGE gel separation and LC-MS analysis.

### Cell cycle analysis

Cells fixed overnight with cold 70% ethanol were digested by RNase at 37°C for 30 min and stained with P.I. or phosphor-Histone H3 prior to flow cytometry analysis with BD FACSVerse^TM^.

### Live-cell imaging, immunofluorescence microscopy

Cells in glass-bottomed dishes were transfected with GFP-H2B and imaged on a Nikon A1 confocal microscope. For immunofluorescence microscopy, cells on coverslips were fixed and permeabilized with 4% paraformaldehyde and cold acetone. Five percent BSA was used as a blocking agent and antibody dilution buffer. For STORM imaging, cells were labeled with secondary antibodies with Alexa Fluor 561/647 dyes (Thermo Fisher Scientific, A31517/A11031) after standard imaging process. The images were acquired on a Nikon N-STORM system.

### Karyotyping

Cells were treated with colchicine for 12 hours and fixed with methanol and acetic acid (3:1 mixture). Cells were then dropped onto slides prior to Giemsa staining, and imaged with an Olympus IX51 microscope.

### Chemical treatments

The following chemicals were used in this study: MG132 (10 mM), colchicine (100 ng/ml), nocodazole (100 ng/ml), S-trityl-L-cysteine (STLC, 5 mM), LY294002 (30 um) and puromycin (2ug/ml).

## SUPPLEMENTARY MATERIALS FIGURES AND TABLES


