# Supplementary material for: PTEN regulates spindle assembly checkpoint timing through MAD1 in interphase
Source: Oncotarget. 2017 Aug 24;8(58):98040–50. doi: 10.18632/oncotarget.20532 (PMC5716712; doi:10.18632/oncotarget.20532)
Supplement: Supplementary file 1 [file oncotarget-08-98040-s001.pdf]

PTEN regulates spindle assembly checkpoint timing through MAD1 in interphase

SUPPLEMENTARY MATERIALS

Extended experiment procedures

Cell culture and antibodies

Cells were cultured in MEM supplemented with 10% FBS. The insect cell line Sf9 was obtained from Invitrogen and characterized by morphological analysis, and cultured in Grace's insect medium (Gibco). Cells were cultured in MEM supplemented with 10% FBS. The human cancer cell line HCT116 was obtained from the American Type Culture Collection (ATCC) in 2009 and maintained according to ATCC recommendations. This cell line was authenticated by Beijing Microread Genetics in November 2016 using STR profiling. The source of the PTEN knock out cell line has been previously described [1].

The following commercial antibodies were used in this study: PTEN (A2B1), PTEN (N19), and MAD1 (H-288) from Santa Cruz; Phosphor-Histone H3 (#3377) from Cell Signaling; Phosphor-Serine (ab9332) and Phosphor-Threonine (ab9337) from Abcam; CREST autoimmune serum (Immunovision HCT-0100); FLAG (M2-3165) and HA (H3163) from Sigma-Aldrich; BUBR1 (A300-995A), CDC20 (A310-396A) and MAD2L1 (A300-301A) from Bethyl; GADPH, and GST antibodies from Sungene Biotech (Tianjin, China).

Other antibodies which were generated in our laboratory using relevant proteins as antigens included PTEN (mouse monoclonal antibody) and MAD1 (mouse polyclonal antibody).

Gene targeting: TALEN MAD1 somatic knock out

The cut site within exon1 was chosen to specifically knockout the MAD1 gene. The genomic recognition sequence of the TALEN left and right arms were CATCTCTCAGCGTGTGG (L) and GGGGCCGAGGTAGAAAT (R), which were spaced by 17bp and anchored by a preceding T base at the -1 position, as this has proved to be optimal for naturally occurring TAL proteins. TALEN vectors for the left and right arms were obtained by one-step ligation using the FastTALE™ TALEN Assembly Kit (SIDANSAI Biotechnology, China) according to the manufacturer's instructions. The two vectors were co-transfected into HCT116 cells with PEI (Polysciences, USA). On the third day after transfection, cells were treated with puromycin (2ug/ml) for 3 days and 48 clones were selected and transferred into two 24-well plates without antibiotics. The MAD1 gene of these clones was analyzed by PCR and DNA sequencing in the 6-well phase. The primers for PCR and DNA sequencing were:

TTGGTCTCCAGGACTTGTCCCAGCA (F)
GGTCGACCTCACGCTCGTAGTTCCT (R)

Transfection, immunoprecipitation, and pull-down assays

Transfection was performed using PEI (Polyethyleneimine, Polysciences, Inc.). Immunoprecipitation was performed as previously described [2].

Cells transfected with S-HA tagged genes were lysed with cold 0.5% NP40 buffer (pH8.0-Tris 10 mM, NaCl 150 mM, 0.5% NP40, protease inhibitors) and incubated with S Tag beads 4°C overnight prior to washing with 0.1% NP40 buffer (pH8.0-Tris 10 mM, NaCl 150 mM, 0.1% NP40) followed by SDS-PAGE gel separation and LC-MS analysis.

Cell cycle analysis

Cells fixed overnight with cold 70% ethanol were digested by RNase at 37°C for 30min and stained with P.I. or phosphor-Histone H3 prior to flow cytometry analysis with BD FACSVerse™.

Live-cell imaging, immunofluorescence microscopy

Cells in glass-bottomed dishes were transfected with GFP-H2B and imaged on a Nikon A1 confocal microscope. For immunofluorescence microscopy, cells on coverslips were fixed and permeabilized with 4% paraformaldehyde and cold acetone. Five percent BSA was used as a blocking agent and antibody dilution buffer. For STORM imaging, cells were labeled with secondary antibodies with Alexa Fluor 561/647 dyes (Thermo Fisher Scientific, A31517/A11031) after standard imaging processing. Images were acquired on a Nikon N-STORM system.

Karyotyping

Cells were treated with colchicine for 12 hours and fixed with methanol and acetic acid (3:1 mixture), and dropped onto slides followed by Giemsa staining, and then imaged with an Olympus IX51 microscope.

Mass spectrometry analysis

Specific gel bands of interest were individually excised and destained with 25 mM NH_4HCO_3 in 50% acetonitrile. Proteins were reduced with 10 mM

Dithiothreitol and alkylated with 50 mM Iodoacetamide. After drying in 100% acetonitrile, the band of interest was digested using sequencing grade trypsin (Promega) at a ratio of 1:100 (enzyme: substrate). Extracted peptides were suspended in 0.1% formic acid and subjected to Nano LC-MS/MS analysis. Peptides were loaded onto a 100 Pm * 10 cm reversed-phase C18 fused silica emitter made in our laboratory, and eluted with 100% acetonitrile and 0.1% formic acid using a linear gradient which ranged from 5% to 32% at a flow rate of 300 nl/min. Data-dependent mass spectra were acquired with an LTQ Orbitrap Elite mass spectrometer (Thermo Fisher Scientific) equipped with a Nano electrospray ion source (Thermo Fisher Scientific). Raw mass spectra files were processed with Proteome Discoverer 1.4 (Thermo Fisher Scientific) and searched with the SEQUEST search engine against the human Uniport database (version 22 2014_02) containing both forward and reverse protein sequences. The precursor ion mass tolerance was set to 10 ppm, and MS/MS tolerance was 0.5 Da. Search parameters were set as follows: trypsin, up to 2 missed cleavages; carbamidomethyl cysteine as a fixed modification; methionine oxidation and phosphorylation of serine, threonine and tyrosine as variable modifications.

Gene Ontology analysis and network visualization

Gene Ontology (GO) enrichment analysis was performed using the Cytoscape plug-in ClueGo. For analysis, over-represented terms in the GO Biological Process were enriched and the GO level was set three to four. The GO term fusion option was selected to reduce redundancy. In order to account for multiple testing, p-values were corrected by Bonferroni correction. Only terms with $p<0.05$ were displayed. The selection criteria for the terms that have associated genes from the uploaded pull down list was set as minimum 3 genes per term and a minimum of 4% of total genes associated with the term for analysis of the PTEN pull down network. The Kappa score was set to 0.4 for analysis of the PTEN network.

Statistics

Statistical significance was determined with the two-tailed Student's t test.

REFERENCES

1. Feng J, Liang J, Li J, Li Y, Liang H, Zhao X, McNutt MA, Yin Y. PTEN Controls the DNA Replication Process through MCM2 in Response to Replicative Stress. Cell Rep. 2015; 13: 1295-303. https://doi.org/10.1016/j.celrep.2015.10.016.
2. Shen WH, Balajee AS, Wang J, Wu H, Eng C, Pandolfi PP, Yin Y. Essential role for nuclear PTEN in maintaining chromosomal integrity. Cell. 2007; 128: 157-70. https://doi.org/10.1016/j.cell.2006.11.042.

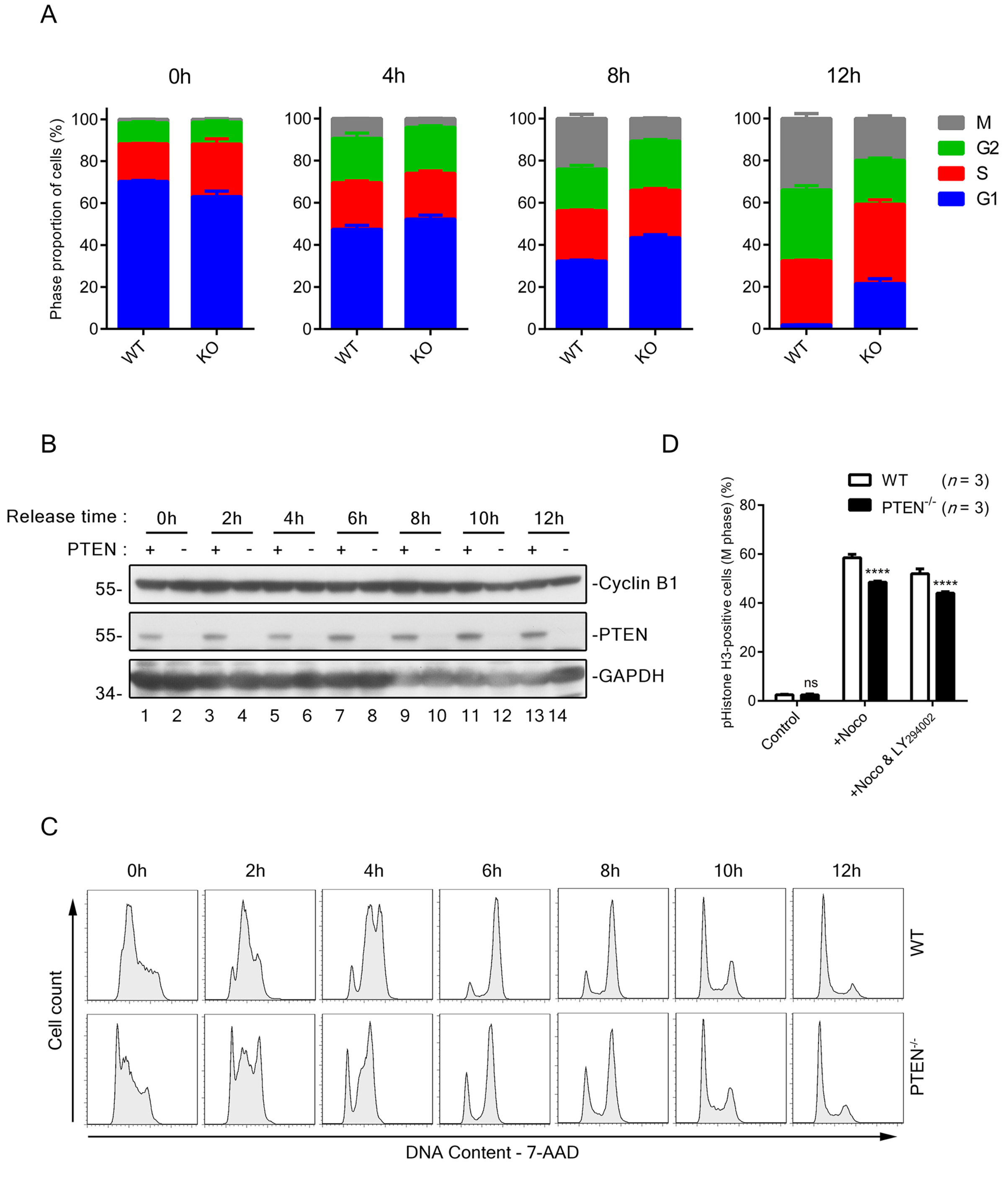

Supplementary Figure 1: PTEN maintains SAC timing, Related to Figure 1. (A) FACS cell cycle profiles of WT and PTEN$^{-/-}$ HCT116 cells. Cells were treated with nocodazole, collected at indicated time points and labeled with pHistone-H3 and PI. Percentages of cells in G2 phase and M phase were analyzed with methods as described in Figure 1D. **(B)** Cyclin B1 profiles of WT and PTEN$^{-/-}$ HCT116 cells. Cells were blocked with thymidine for 12 hours and released for indicated time, and levels of Cyclin B1 were evaluated by blotting. **(C)** FACS profiles of 7-AAD stained WT and PTEN$^{-/-}$ HCT116 cells. Cells were blocked with thymidine for 12 hours and released for indicated times. **(D)** FACS profiles of pHistone-H3 labeled (M phase) WT and PTEN$^{-/-}$ HCT116 cells. Cells were treated with indicated drugs for 12 hours and labeled with pHistone-H3. *n*, number of times each independent FACS assays was performed (~10 000 cells were analyzed each time). Values in all data are presented as mean ± SD. Statistical significance was determined with the unpaired t-test, ns - non-significant, ****P<0.0001.

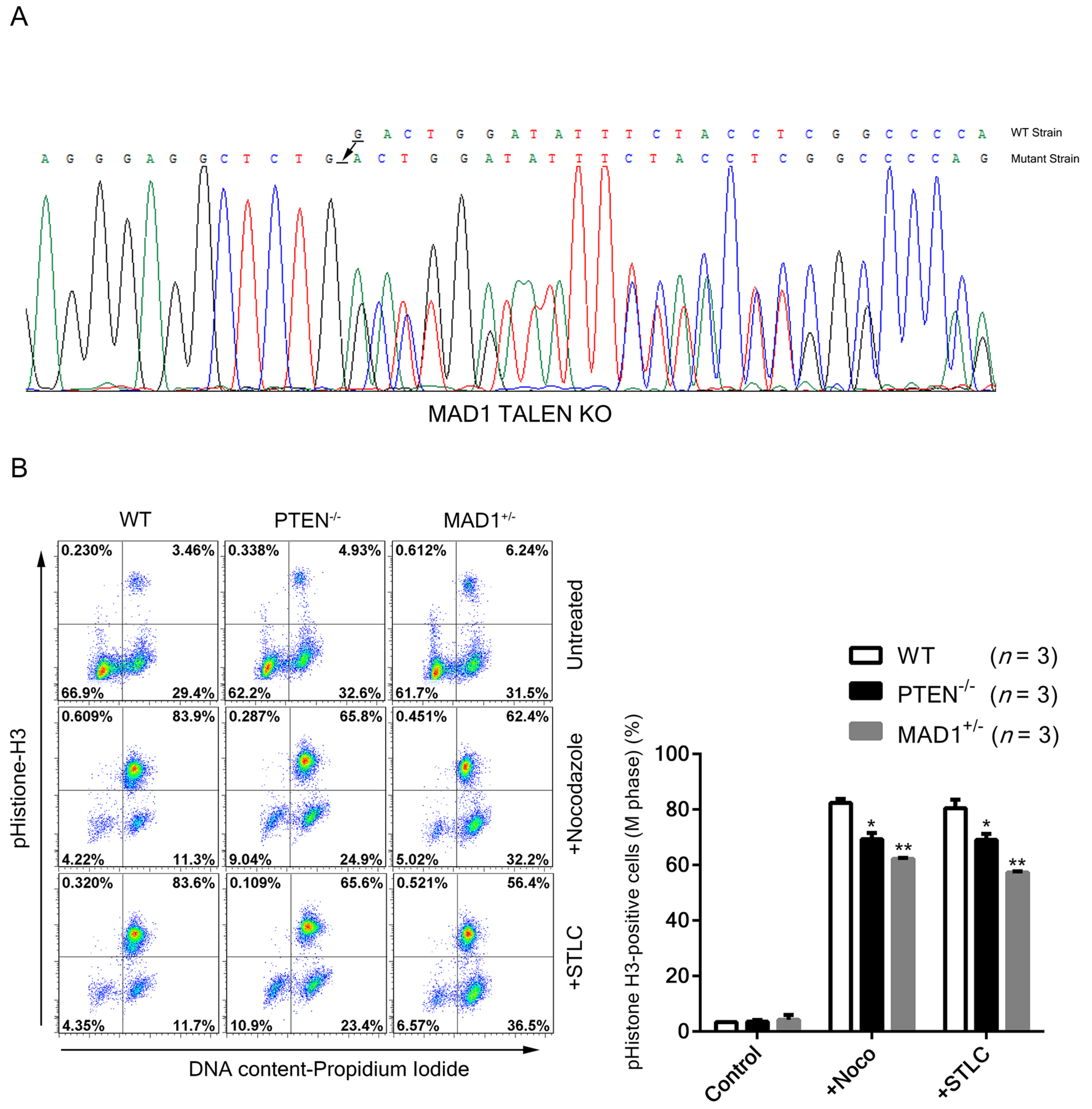

Supplementary Figure 2: Somatic HCT116 MAD1$^{+/-}$ leads to spindle assembly checkpoint override, Related to Figure 3. (A) Establishment of MAD1 somatic knockout systems in HCT116 cells using the TALEN approach. Exon 1 of the MAD1 gene was targeted to generate a stop codon (indicated as black frames) as described in Methods. Sequence alignments comparing WT and TALEN mutated MAD1 in HCT116 cells are shown. **(B)** FACS profiles of pHistone-H3 labeled (M phase) WT, PTEN$^{-/-}$ and MAD1$^{+/-}$ HCT116 cells. Cells were treated with indicated drugs for 12 hours and labeled with pHistone-H3. *n*, number of times each independent FACS assay was performed (~10 000 cells were analyzed each time). All data are presented as mean ± SD. Statistical significance was determined with the unpaired t-test, *P<0.05, **P<0.01.

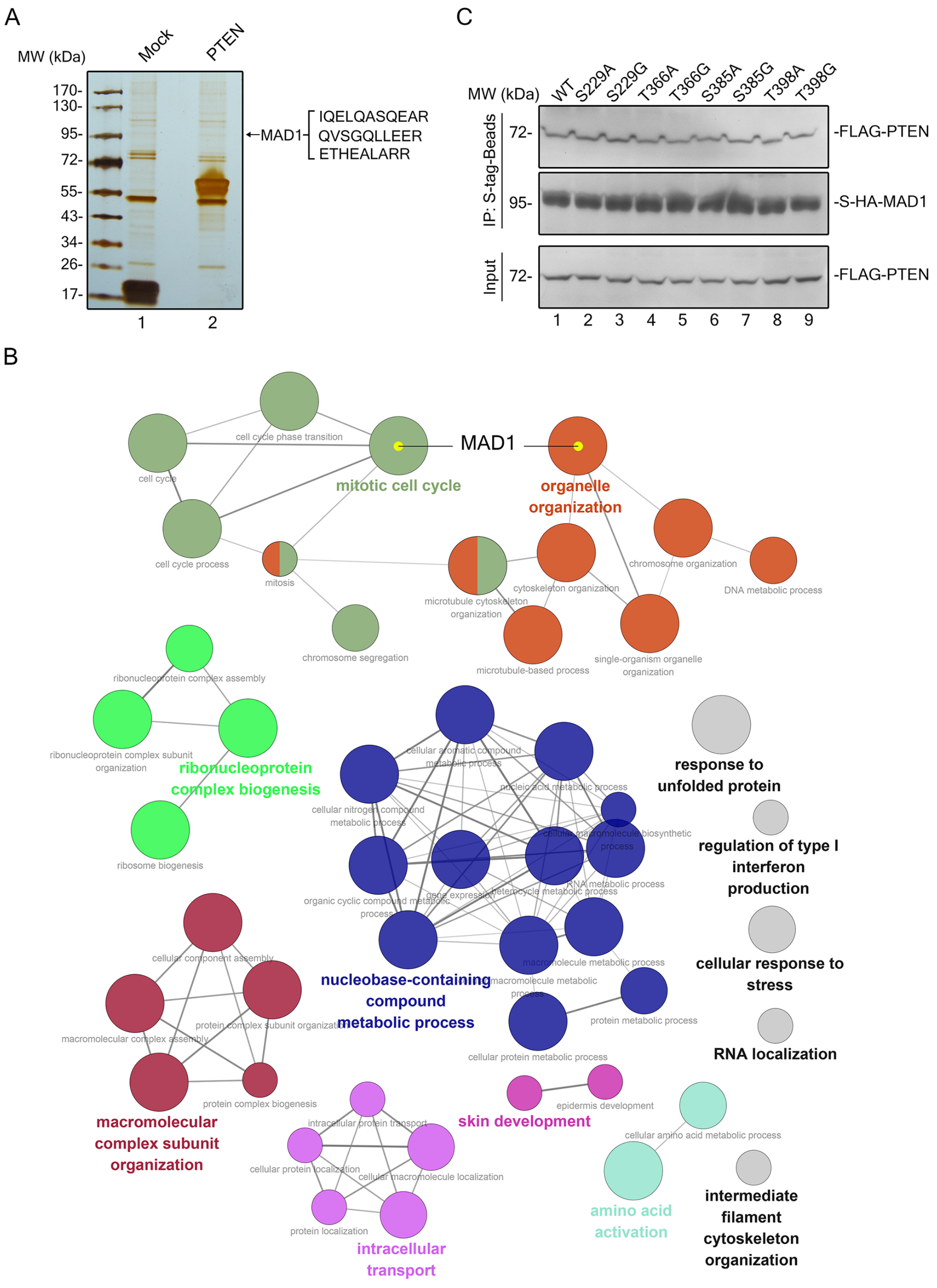

Supplementary Figure 3: Candidates revealed by PTEN pull down assay, Related to Figure 3. (A) S-HA double tagged PTEN or mock plasmid was co-transfected with ubiquitin into HEK293T cells and treated with MG132 prior to pull down with S-tag beads followed by assay with LC-MS. MAD1 peptides were identified. **(B)** Functionally grouped Gene Ontology annotation network of 1082 PTEN pull down genes. Nodes represent enriched terms, and node sizes indicate term enrichment significance after Bonferroni correction. Edges between two nodes represent initial genes shared by two enriched GO terms, and thickness of the edges is based on their kappa score. The calculated kappa score is also used for defining functional groups, which are displayed in the same color used for the nodes. Ungrouped terms are shown in gray. The most significant enriched GO term for each group is shown in bold face. In this analysis, MAD1was drawn in the network and linked with the GO terms it contributes to. **(C)** S tag pull down analysis. Exogenous S-HA-MAD1, FLAG-PTEN or its mutants were transfected into HCT116 cells. MAD1 was pulled down by S-tag beads and detected with a FLAG antibody for PTEN.

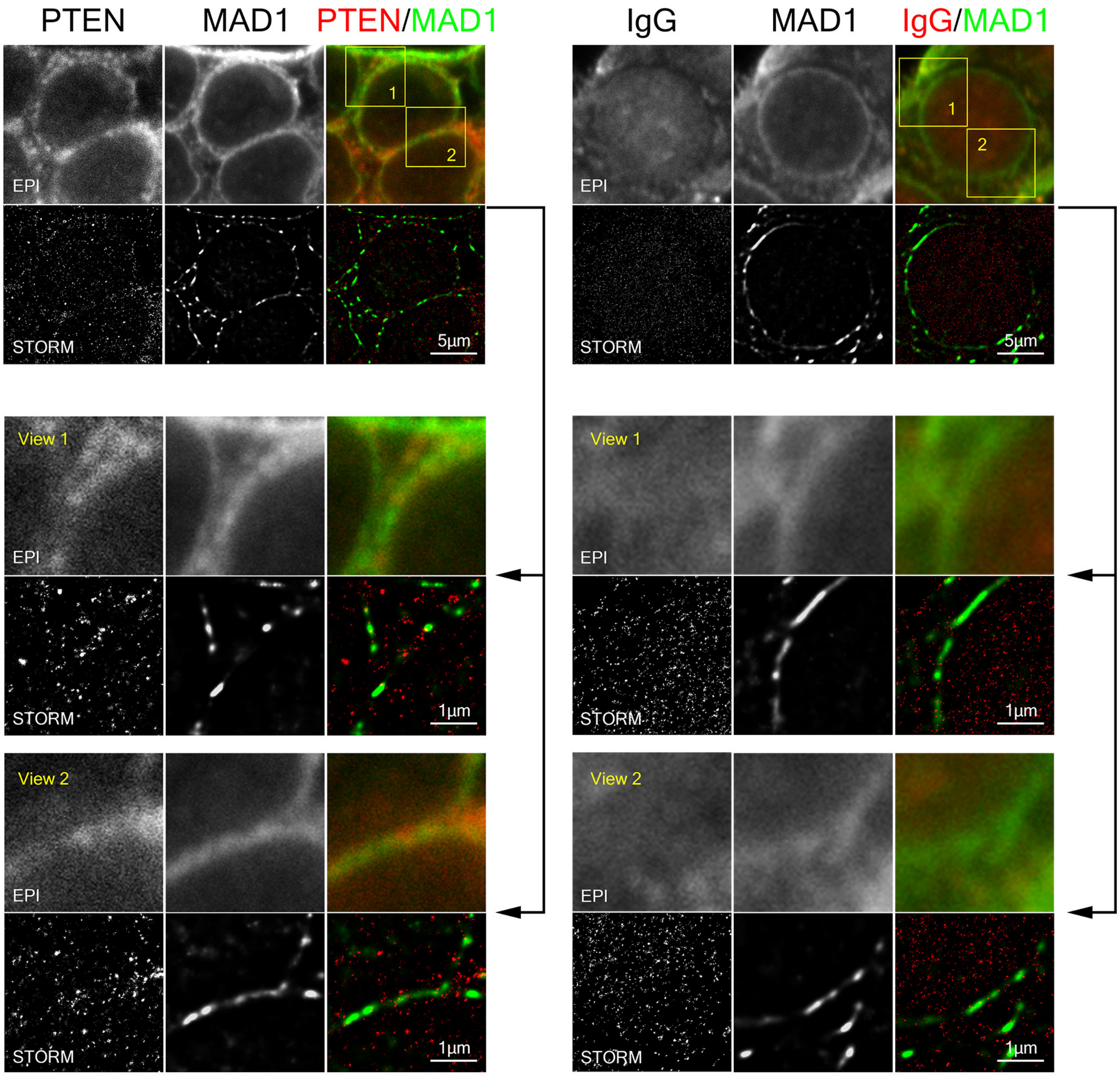

Supplementary Figure 4: PTEN co-localizes with MAD1 at the nuclear envelope, Related to Figure 4. STORM super resolution and EPI image of PTEN and MAD1. Left panel, fixed HCT116 cells were stained for PTEN (green) and MAD1 (red). Single z sections (interphase cells) are shown. Right panel, negative control with IgG. The scale bars are indicated on the images. IgG, immunoglobulin G.

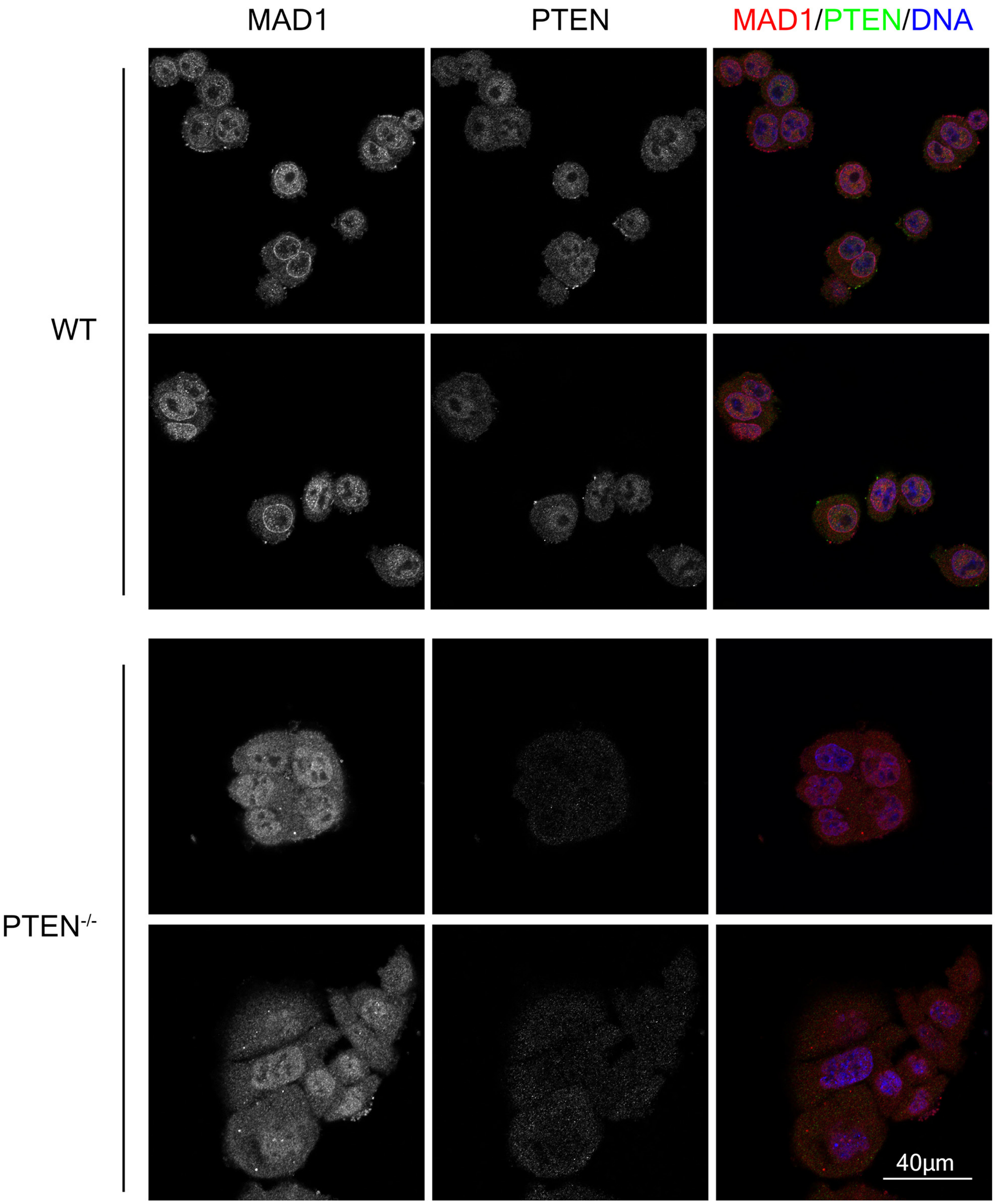

Supplementary Figure 5: NPC localization ability of MAD1 was reduced in PTEN$^{-/-}$ HCT116 cells, Related to Figure 4. Immuno-fluorescence staining of fixed WT and PTEN$^{-/-}$ HCT116 cells. Cells were stained for MAD1 (red), PTEN (green) and DNA (blue). Single z sections are shown with scale bars in 40 µm.

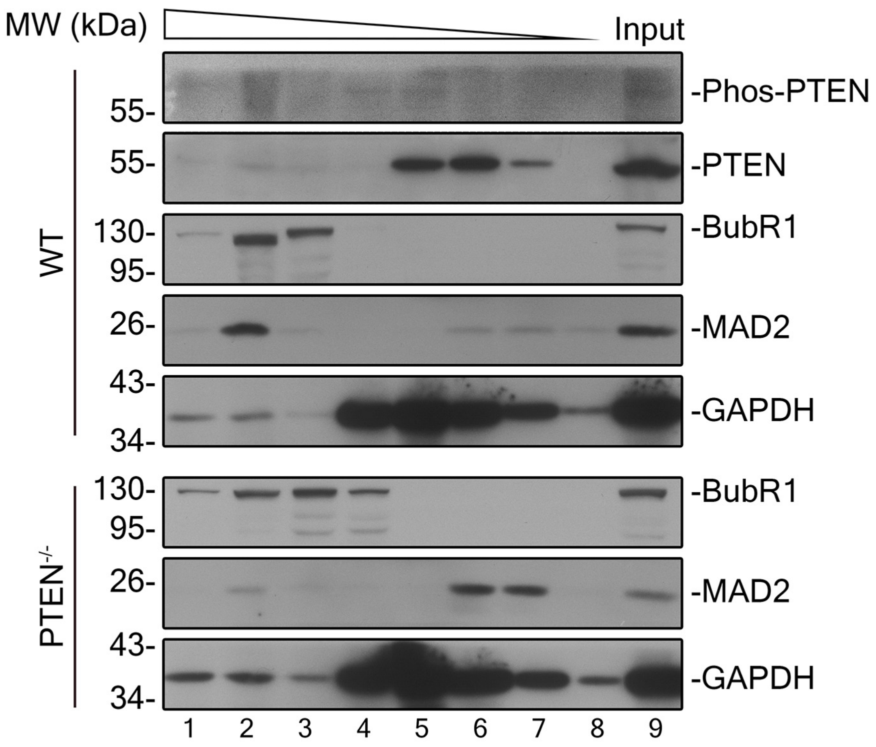

Supplementary Figure 6: Gel filtration chromatography of WT and PTEN$^{-/-}$ HCT116 cells, Related to Figure 5. Indicated MCC components were blotted in interphase extracts of indicated cell lines after gel filtration chromatography. Phosphorylated PTEN was pulled down by PTEN antibody and blotted with phosphorylated Ser/Thr general antibody.
